# Evaluation of the Osteogenic Potential of Periodontal Ligament and Tooth-Derived Substrates Effect on Osteogenesis: An In Vitro Pilot Study

**DOI:** 10.1007/s12663-024-02311-4

**Published:** 2024-08-21

**Authors:** Tiago Pinto Carvalho, Thiago Resck, Davi Kirsch, Marcelo Sperandio, Marcelo Henrique Napimoga, Júlio César Joly, Gustavo Vicentis Oliveira Fernandes, Daiane Cristina Peruzzo

**Affiliations:** 1 Faculty of Dentistry, ULBRA, Canoas, Brazil; 2 Faculty of Dentistry, São Leopoldo Mandic Research Institute, Campinas, Brazil; 3 A. T. Still University - Missouri School of Dentistry & Oral Health, 1500 Park Ave, St. Louis, MO 63104 USA

**Keywords:** Dentin, Periodontal ligament, Ground tooth, Biomaterials, Bone, Osteogenesis

## Abstract

**Objective:**

The goal of this in vitro pilot study was to compare the osteogenic potential of different ground human tooth preparations.

**Methods:**

Twelve maxillary third molars were included and divided into four groups: FT (full tooth), containing all dental tissues (enamel, dentin, pulp, cementum, and periodontal ligament); WE, without enamel; WPL, without periodontal ligament; and WEPL, without enamel and periodontal ligament. The teeth were ground to macroscopically homogeneous particles. The new bone formation was the primary variable evaluated. Pre-osteoblastic cells were incubated with protein extracted from this substrate to verify their osteoinductive potential. Cell proliferation, viability assays, mRNA expression of type I Collagen (COL-I), RUNX-2, BMP-2, and mineral nodules were assessed to achieve the main outcome. Data were analyzed using two-way ANOVA and Tukey tests, considering alpha = 5%.

**Results:**

The FT group had the lowest proliferation cell rates, whereas WEPL had the highest rates (*p* < 0.05). Moreover, there was an increased gene expression of all genes studied in the WEPL group and a greater formation of phosphate nodules (*p* < 0.05).

**Conclusion:**

The dental substrate without enamel and periodontal ligament (WEPL) showed better and improved results than the other groups, revealing promising osteogenic potential for use as a biomaterial for grafting.

## Introduction

Tooth loss and surgical wound healing lead to alveolar atrophy secondary to losing local stimulus from masticatory forces. This reduction represents 30–50% of the total volume of the alveolus in the 1st year and reaches 60% in 5 years [1, 2]. Less bone resorption is observed when alveolar preservation procedures are performed using fresh alveolus bone grafting materials, which in turn reduces the morbidity of subsequent procedures for dental rehabilitation [1, 3, 4].

Tissue engineering is a novel concept in regenerative medicine and dentistry [5]. Using a combination of stem cells, scaffolds, and growth factors, tissue engineering offers promising results for regenerating damaged or lost tissues [5, 6]. Autologous bone grafting is the gold standard but is also associated with high resorption rates and morbidity [7]. Organic dentin matrix contains macromolecules encountered in connective tissues. Odontoblasts synthesize this matrix and are a rich source of growth factors and bioactive molecules required for dentinogenesis [8]. Dentin collagen forms a compact and crosslinked scaffold in which mineral crystals deposit [9] and contains transforming growth factor-b (TGF-b1), insulin-like growth factor (IGF), and bone morphogenetic proteins (BMPs) as well as some angiogenic growth factors [5].

In a set of experiments performed in vitro*,* dentin protein extracts affect the proliferation and differentiation of osteoprogenitor cells, suggesting that TGF-*β* and other factors in dentin may regulate cellular behavior and influence the development, remodeling, and regeneration of mineralized tissues [10]. Some studies have reported using dentin as bone regeneration material in various forms: crushed, in blocks, or demineralized [11–13], while others used the whole tooth as substrate [14], showing promising results in filling the extraction socket. Considering the promising role of human teeth as biomaterials, studies are needed to clarify their bioactivity, as there still remains a plethora of possible preparation methods for human teeth as graft material and various potential forms of management strategies to explore the stimulatory effect of each tissue, for example, enamel, cementum, dentin, pulp, and periodontal ligament on bone neoformation.

Thus, the aim of this pilot study was to compare the osteogenic potential of different tooth preparations of ground human teeth.

## Materials and Methods

This study was submitted and approved by the Research Ethics Committee of the São Leopoldo Mandic Research Institute (SP, Brazil) under registration number #1,963,827. Before inclusion, all patients signed the informed consent, permitting the inclusion of the tooth. The recruitment period and study duration were from April 2022 to July 2023.

### Sample Characteristics and Eligibility Criteria

For the inclusion of the patients, it was followed: (1) 12 freshly extracted human third molars (one upper or one lower per patient) with fully formed roots were selected from six healthy individuals (without any systemic condition); (2) tooth referred for extraction due to orthodontic reasons or prevention of adjacent tooth resorption; (3) patients ≥ 18 years old; and (4) with completely included and impacted tooth. It was excluded: (1) pregnant patients; (2) smokers; (3) patients with any systemic condition other than healthy; or (4) extraction of not impacted/totally included tooth.

The extractions were performed by the same professional under local anesthesia (2% lidocaine and epinephrine at 1:100,000). The soft tissues surrounding the tooth were incised and removed to facilitate luxation and subsequent extraction. There was no section in the tooth, which was a motive for exclusion. The extracted tooth was abundantly rinsed with saline solution to remove blood and debris.

### Groups

The teeth were divided into four test groups (*n* = 3) and a control group: Group 1. Control Group—no tooth-derived material was used—the cell culture was only supplemented with culture medium to keep the pre-osteoblastic cells viable; Group 2. FT (full tooth): the tooth was wholly preserved: enamel, dentin, periodontal ligament, cementum, and pulp; Group 3. WE (without enamel): enamel was removed using new diamond burs at high speed and water cooling; Group 4. WPL (without periodontal ligament): only the periodontal ligament was removed using periodontal curettes; and Group 5. WEPL (without enamel and periodontal ligament): enamel and periodontal ligament were removed.

After extraction, the teeth were randomly distributed (head or tail) by the groups until three teeth were achieved. They were immediately ground (according to the group distribution) using a manual bone-crushing pestle until the granules were visually (macroscopically) homogeneous.

### Protein Extraction and Quantification

After grinding, the teeth were deposited in 15-ml polystyrene test tubes containing 1.5 mL of RIPA lysis buffer solution (Thermo Scientific, USA), containing 50 mM Tris HCl (pH 7.4), 150 mM NaCl, 1 mM EDTA, 1% Triton X-100, 1% sodium deoxycholate and 0.1% sodium dodecyl sulfate (SDS), plus a protease inhibitor cocktail (Sigma, St. Louis, MO, USA) at the final concentration of 1%, for each sample. Each tube was then shaken on a shaker (Phoenix AP 56) for 30 s to homogenize the elements contained in the samples.

The tubes were centrifuged (Eppendorf 5804 R) at 4 °C and 156G for 15 min, resulting in a three-phase solution. The supernatant corresponding to the protein extracts was pipetted out and transferred to new polystyrene tubes (Eppendorf, USA), duly identified.

The samples' total protein quantification was performed using the BCA Protein Assay kit (Thermo Scientific, USA), following the manufacturer's guidelines. The different concentrations of protein extracts (10 and 100 ng/nL) of the groups were added to the cell culture, and the results were compared to the control group.

### Cell Cultures

The mouse’s pre-osteoblastic cell line (MC3T3-E1) used in this study was obtained from the American Type Culture Collection (ATCC, VC, USA). Pre-osteoblastic cells were cultured in alpha modification of minimum essential medium (α-MEM) supplemented with 10% fetal bovine serum (Cultilab®, Campinas, SP, Brazil) and 100 U/mL penicillin and 100 μg/mL streptomycin (Sigma, St. Louis, Missouri, U.S.A.).

All procedures were performed in a laminar flow hood to maintain the sterility of the materials and substances used for cell culture. The cells were kept in an incubator at 37 °C in a humid atmosphere containing 95% air and 5% carbon dioxide. The culture medium was changed every 2–3 days, and the culture progression was evaluated using phase microscopy in cultures grown on polystyrene. The concentrations tested in the present study were obtained from a concentration-effect curve.

### Cell Proliferation Assay

For the cell proliferation assay, the Trypan blue vital exclusion method was used. The cells were cultured to a density of 110 cells/mm^2^ in 24-well plates and exposed to different protein extracts. After 1, 3, and 7 days, the cultures were enzymatically removed from the plates, and the cell pellet resulting from the centrifugation was suspended in 1 ml of medium. 10 μL of the cell suspension was removed, and 10 μL of Trypan blue was added to it. 1 μL of this suspension was then placed onto a hemocytometer (Neubauer-Fisher Scientific chamber, Pittsburgh, PA, USA) for manual counting under an inverted phase microscope (Nikon, Eclipse TS100). The data obtained were expressed in the number of cells × 10^4^.

### Cytotoxicity Assay (MTT)

Cell cultures were tested for cell viability using the MTT assay. In the cytotoxicity assay, cells were cultured at a density of 110 cells/mm^2^. After 1, 3, and 7 days of exposure to protein extracts from the different groups, the culture medium was removed and a new medium containing MTT (5 mg/mL, Sigma-Aldrich, U.S.A.) was added; cell cultures were incubated for 3 h at 37 °C. After this period, 100 μl of DMSO (Dimethylsulfoxide, LGC, São Paulo, Brazil) was added for 15 min at room temperature. The solubilized crystals were quantified in an ELX800 microplate reader (Biotek Instruments, Inc.) at 590 nm. The data were expressed as absorbance.

### mRNA Expression of Genes Involved in Osteogenic Differentiation

The expression of genes encoding type I collagen (COL1), runt-related transcription factor 2 (RUNX-2), and bone morphogenetic protein 2 (BMP-2) was assessed by the real-time polymerase chain reaction (RT-PCR). The MC3T3-E1 cells were cultured in 24-well plates and treated with protein extracts for 1 and 3 days. Total RNA was extracted to make the cDNA using the Platus Transcriber Rnase H-cDNA First Strand kit (Sinapse inc).

Real-time PCR reactions were performed on a 7500 Fast Real-Time PCR System (Applied Biosystems) using the SYBR Green/ROX qPCR reagent (Thermo Fisher) as the detection system. The sequence of the primers used is listed in Table 1. Expression of the COL1, RUNX-2, and BMP-2 genes was normalized in relation to the endogenous gene GAPDH and calculated using the 2^−ΔΔCt^ method. The values ​​ were expressed in arbitrary units (AU).

**Table 1:** Primer sequences used in this study

Primer	Sequence
Primer GAPDH forward	TGGCCTCCAAGGAGTAAGAAAC
Primer GAPDH reverse	TGGAAATTGTGAGGGAGATGCT
Primer RUNX-2 forward	TGCCTCCGCTGTTATGAAAA
Primer RUNX-2 reverse	CTGTTATGGTCAAGGTGAAACT
Primer Collagen tipo 1 forward	ACAAGGTGACAGAGGCATAAAGG
Primer Collagen tipo 1 reverse	GCCTGCAGGACCTGAAGCT
Primer BMP-2 forward	TGTCTTCTAGTGTTGCTGCTTC
Primer BMP-2 reverse	CTCAACTCAAATTCGCTGAGGAC

### Von Kossa Staining

The pre-osteoblastic cells were cultured with the protein extract for 14 days, and the culture medium/protein extract was changed every 2–3 days. After 14 days, the culture medium was removed, and 2 ml of silver nitrate (AgNO3) was placed in each well for 20 min. The wells were then rinsed with distilled water, and 2 ml of hydroquinone (C6H4 (OH) 2) was added for 2 min. 2 ml of thiosulfate (S2O3−2) was then added for 2 min. The samples were rinsed with distilled water and prepared for analysis under light microscopy (Nikon Eclipse E800, Japan). Phosphate deposits were identified by the presence of black-colored deposits by means of macroscopic photos converted into binary data. Image analysis was performed using the ImageJ software (NIH).

### Statistical Analysis

All experiments were performed in triplicate. The mean values and standard deviations were compared using a two-way analysis of variance (ANOVA) and Tukey/Dunnett-Sidak tests at a significance level of 5% (*p* < 0.05). The analyses were performed on GraphPad Prism 9.5.1 (GraphPad Software, LLC).

## Results

The teeth extracted, two per patient (12 molar/6 patients), were randomly distributed: Group 1 (control): two upper molars and one lower molar; Group 2: one upper molar and two lower molars; Group 3: one upper molar and two lower molars; and Group 4: two upper molars and one lower molar.

### Cell Proliferation

**Fig. 1:**
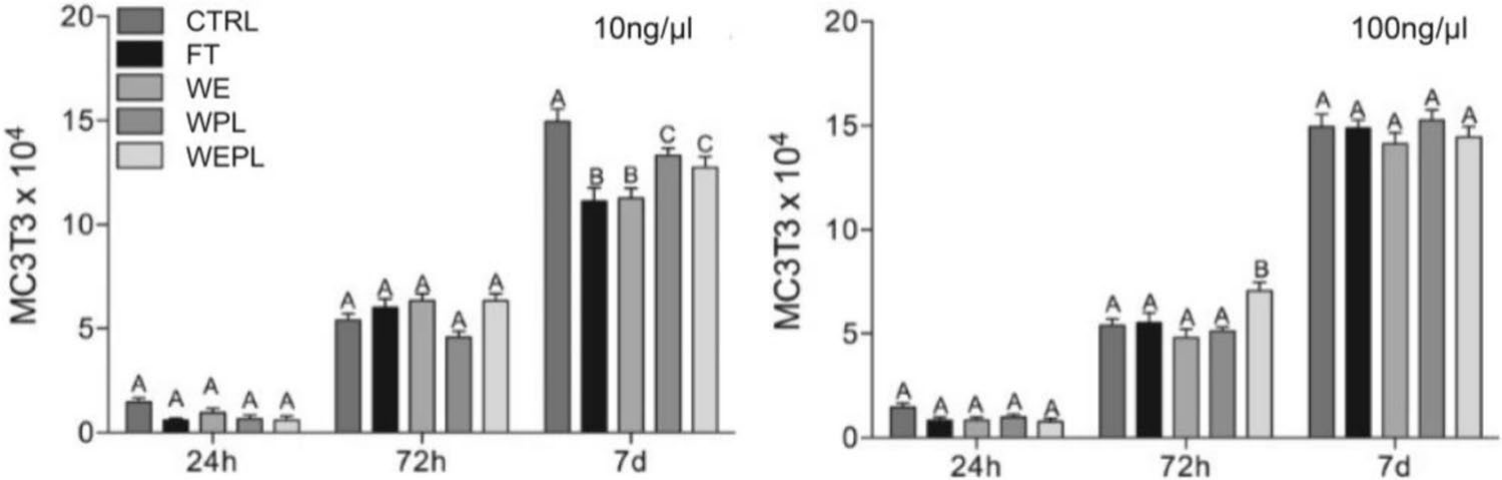
Means and standard deviation of cell proliferation of cell cultures treated with protein extract from different groups over 24, 72 h, and 7 days. Different capital letters indicate a significant difference (*p* < 0.05) between groups within the same time period

Pre-osteoblastic cell proliferation treated with protein extract from different human tooth substrates at 10 and 100 ng/µl in relation to the control group (untreated) is shown in Fig. 1. The concentration of 10 ng/µL caused no difference in cell proliferation between the different substrates at 24 and 72 h. However, at 7 days, the groups in which the periodontal ligament was presented (FT and WE) had lower cell proliferation (*p* < 0.05). At the concentration of 100 ng/µL, a significant increase in proliferation was observed in the group WEPL at 3 days (*p* < 0.05), and no significant difference was observed between groups in the remaining study periods (*p* > 0.05).

### Cytotoxicity Assay (MTT)

**Fig. 2:**
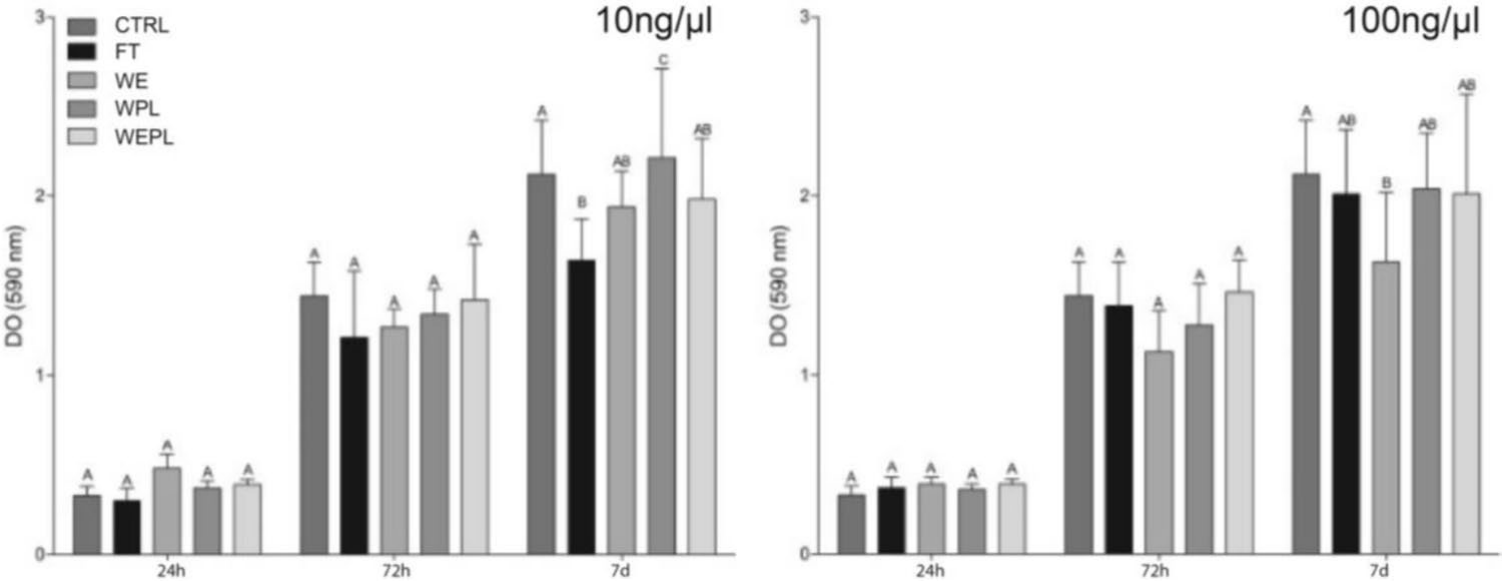
Means and standard deviation of cell viability for the different groups at 24, 72 h, and 7 days. Different capital letters indicate a significant difference between groups at the same time (Anova Dunnett and Sidak, alpha of 5%, tests performed in relation to the control group)

Cell viability in the presence of protein extract from different tooth substrates (Fig. 2) showed the lowest viability rates for the FT group, while the group without periodontal ligament (WPL) had the highest rates (*p* < 0.05) at 10 ng/µL. At 100 ng/µL and 72 h, the group without enamel and with periodontal ligament (WEPL) showed significantly lower rates (p < 0.05).

### mRNA Expression of COL-I, RUNX-2, and BMP-2

**Fig. 3:**
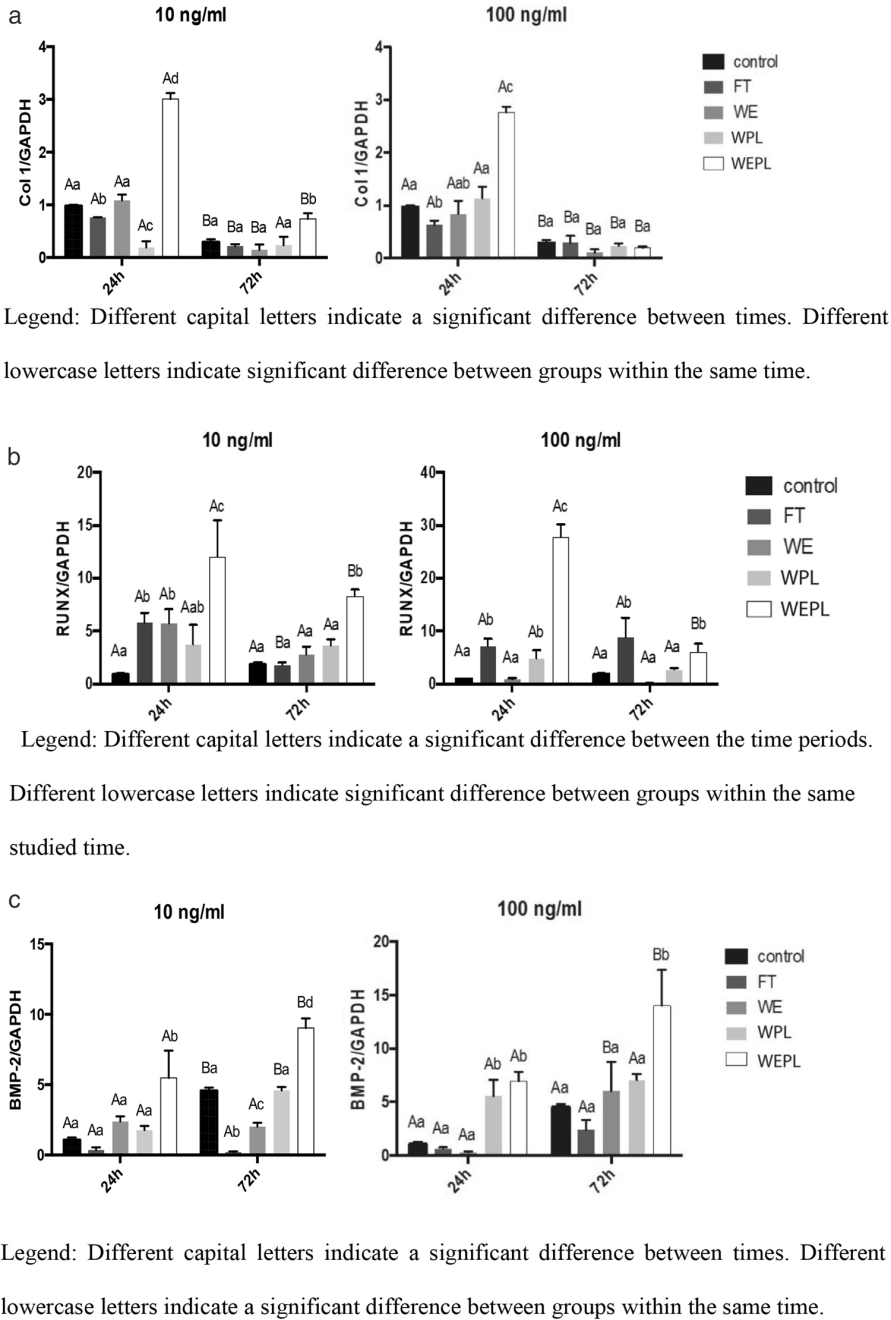
**a** Means and standard deviation for type I collagen mRNA expression in osteoblasts treated with protein extracts from different tooth substrates in 24 and 72 h. Different capital letters indicate a significant difference between times; different lowercase letters indicate a significant difference between groups within the same time; **b** Means and standard deviation of RUNX-2 gene expression in pre-osteoblastic cells exposed to protein extracts from different tooth substrates in 24 and 72 h. Different capital letters indicate a significant difference between the time periods; different lowercase letters indicate a significant difference between groups within the same studied time; c. Means and standard deviation of BMP-2 gene expression in cultures of pre-osteoblastic cells exposed to protein extracts from different tooth substrates at 24 and 72 h. Different capital letters indicate a significant difference between times; different lowercase letters indicate a significant difference between groups within the same time

As for COL-I, the highest gene expression at 10 ng/µL was observed in the WEPL group (*p* < 0.05), both in 24 and 72 h. At 100 ng/µL, gene expression was significantly lower for the FT group and significantly higher for the WEPL group when compared to the control group (*p* < 0.05) (Fig. 3a).

Regarding RUNX-2 expression at 10 ng/µl, a significant difference (*p* < 0.05) was observed in the FT, WE, and WEPL groups at 24 h compared to the control, with WEPL showing the highest expression in relation to the remaining groups, as shown in Fig. 3b. At 100 ng/µl and 24 h, a significant upregulation (*p* < 0.05) was observed for the WEPL groups compared to the control. At 72 h, a significant upregulation (*p* < 0.05) was observed for the FT and WEPL groups, with no difference.

Regarding BMP-2 at 10 ng/µl, a significant increase (*p* < 0.05) was observed only in the WEPL group at 24 h in relation to the remaining groups. At 72 h, a significant difference (*p* < 0.05) was observed for the FT, WE, and WEPL groups in relation to the control, with lower expression in the FT group and higher in the WELP group, as shown in Fig. 3c.

At 100 ng/µl and 24 h, greater expression (*p* < 0.05) was observed for the WPL and WEPL groups compared to the remaining groups. At 72 h, only the WEPL group showed higher expression (*p* < 0.05) than the remaining groups.

### Mineral Nodules Formation

**Fig. 4:**
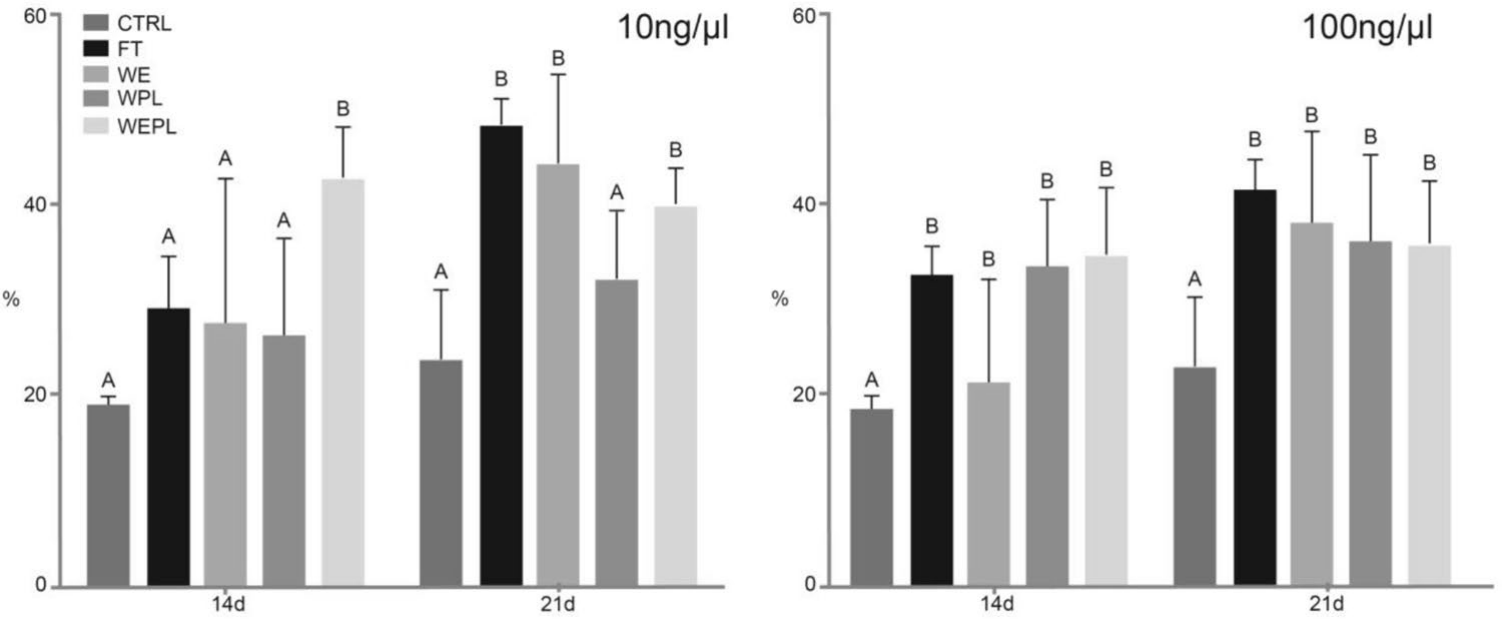
Means and standard deviation of the mineralization assay at different concentrations of protein extract. Different capital letters indicate a significant difference between groups

At 10 ng/µL and 14 days, the WEPL group showed the greater formation of phosphate nodules when compared to the remaining groups (*p* < 0.05), whereas, at 21 days, FT, WE, and WEPL had the highest mineral content (*p* < 0.05) than the WPL and control group. At 100 ng/µL and regardless of time, all groups showed greater mineral content than the control group (*p* < 0.05), as shown in Fig. 4.

## Discussion

From the biological point of view, autologous bone remains the ideal filling material for extraction sockets due to its osteogenic, osteoinductive, and osteoconductive properties [7, 15], although the use of autologous bone is riddled with issues such as morbidity to the donor area, limited availability of graft volume, and additional operating time for harvesting autologous bone, which has led to an increasing demand for bone substitutes [4, 5, 16]. Dentin and bone are known to have similar organic and inorganic structures [17]. Recent studies have focused on dentin as a potential bone substitute in different models of alveolar defects [18]. However, due to differences in how autologous teeth are used as grafting material [10, 13], this study sought to compare the osteogenic potential of different preparations of ground human teeth in vitro*.* In general, dentin modulated the proliferation of osteoblastic cells in vitro, stimulating the expression of genes involved with osteogenic differentiation, as well as promoting mineralization of the extracellular matrix. The results of the present study suggest that dentinal tissues can be considered alternative graft materials to autologous bone.

In a pilot study carried out by our group (unpublished data), different concentrations of total protein extracts from crushed teeth were tested. In view of the results obtained therein, the lowest and highest concentrations (10 and 100 ng/µL) were selected for the present study. Cell proliferation and viability were negatively affected by the presence of periodontal ligament, while dentin, due probably to its physiological similarity with bone, showed superior results. This is in line with the findings of some studies, which indicate the presence of biostimulating factors of mineralization such as BMP and inorganic components, for example, hydroxyapatite, beta-tricalcium phosphate, ammonium calcium phosphate, and octacalcium phosphate, in dentin [19].

Quantification of type I collagen, Runx-2, and BMP also tended to increase when enamel and periodontal ligament tissues were absent, corroborating the findings from a previous in vivo study that demonstrated good biocompatibility of processed dentin in the femur of rats [20]. By contrast, the findings reported herein do not corroborate the hypotheses that the organic constituents in the periodontal ligament would induce differentiation of mesenchymal cells [19]. The favorable outcomes demonstrated in the absence of periodontal ligament corroborate some findings, highlighting that such tissue substrate is resistant to mineralization [21, 22].

As for the capacity of pre-osteoblastic cells to form calcified nodules under the stimulation of protein extracts from different preparations of autologous tooth tissues, all groups showed superior results to those observed in the control group, and mineralization content is an indication of the osteogenic potential of ground autologous teeth. It is important to highlight that in all the preparations reported herein, the cementum was presented, even in the group where the periodontal ligament was removed, because it would not be possible to ensure in a clinical setting that the cement was completely removed using periodontal curettes only. Though avascular, cement contains approximately 50% organic material (90% type I collagen) and 50% inorganic matrix (60% hydroxyapatite) [22].

Studies have demonstrated the potential of cement to stimulate undifferentiated mesenchymal cells [23]. In the present study, the method selected to prepare the protein eluate could not ascertain cement's macroscopical presence or absence. Thus, a possible explanation for the groups with no ligament showing a greater biostimulatory potential may be that, for these groups, removal of the ligament promoted a considerable exposure of cementum, which in turn translated into osteoblast activation.

In general, the group in which the enamel and periodontal ligament were absent (WELP) revealed the most promising results, which agrees with most studies that used the dentin matrix exclusively as filling material [19, 20]. The findings from the in vitro assays presented herein warrant further investigation using preparations that exclude enamel and periodontal ligament, thus prioritizing dentin and its organic compounds as a stimulus for bone neoformation [24].

Extracted teeth are usually discarded, though they could be useful as autologous graft material for the extraction socket. This could minimize the incidence of bony defects following exodontia at a low cost and bypass the risk of immunological reactions compared to alveolar preservation based on xenografts.

As a limitation of this study, the low number of teeth involved (pilot study) and the lack of a group testing the osteogenic potential of pulp tissue can be pointed out, which should be observed in future studies.

## Conclusion

Even though it was an in vitro pilot study and observed its limitations, it is possible to conclude that dentin and pulp tissue substrates (without enamel and without periodontal ligament) demonstrated promising osteogenic potential in vitro. More studies are recommended to be developed, observing the limitations pointed out.
